# A novel prognostic biomarker *LCP2* correlates with metastatic melanoma-infiltrating CD8^+^ T cells

**DOI:** 10.1038/s41598-021-88676-9

**Published:** 2021-04-28

**Authors:** Zijun Wang, Mou Peng

**Affiliations:** 1 Institute of Dermatology, Chinese Academy of Medical Sciences and Peking Union Medical College, Nanjing, 210042 Jiangsu China; 2 Department of Dermatology, The Second Xiangya Hospital of Central South University, Changsha, 410011 Hunan China; 3 Department of Urology, The Second Xiangya Hospital of Central South University, Changsha, 410011 Hunan China; 4 Department of Microbiology and Immunology, Albert Einstein College of Medicine, Bronx, NY 10461 USA

**Keywords:** Biomarkers, Medical research

## Abstract

Lymphocyte cytosolic protein 2 (*LCP2*) is one of the SLP-76 family of adapters, which are critical intermediates in signal cascades downstream of several receptors. *LCP2* regulates immunoreceptor signaling (such as T-cell receptors) and is also required for integrin signaling in neutrophils and platelets. However, the role of *LCP2* in the tumor microenvironment is still unknown. In this study, we found a significant increase of mRNA and protein expression of *LCP2* in metastatic skin cutaneous melanoma compared to normal skin. The upregulation of *LCP2* was associated with good overall survival of patients with metastatic skin cutaneous melanoma, who received pharmacotherapy and radiation. GSEA signaling pathways analysis showed that *LCP2* was involved in multiple pathways of immune response and correlation analysis revealed *LCP2* was positively correlated with molecules in TCR signaling and 11 immune checkpoints, while *LCP2* negatively correlated with 2 immune checkpoints in the metastatic skin cutaneous melanoma. According to the different expressions of *LCP2*, high *LCP2* expression was positively correlated with more tumor-infiltrating CD8^+^ T cells. Furthermore, Kaplan–Meier plot indicated that *LCP2* acted as a prognostic biomarker for progression-free survival of patients with metastatic skin cutaneous melanoma receiving anti-PD1 immunotherapy. In conclusion, our results integrated both the expression and function of *LCP2* in melanoma using multiple tools, shedding light on the potential role of *LCP2* in melanoma, and suggesting *LCP2* serves as a prognostic biomarker and therapeutic target in anti-tumor immunity.

## Introduction

Studies of immune checkpoints, such as PD-1 and CTLA4, provide novel immunomodulatory targets to treat malignant tumors^1^. Immune checkpoint inhibitors have revolutionized the treatment for metastatic melanoma and substantially improved patients’ survival rate. Tumor-infiltrating immune cells play essential roles in affecting disease prognosis and are indicators of anti-tumor responses in patients with metastatic melanoma receiving immune checkpoint inhibitors^2^. Although some prognostic biomarkers^3–5^ for overall survival (OS) and response have been mentioned in melanoma, most of them are not sensitive and/or specific enough to be used for evaluating the infiltrating-immune cells based anti-tumor response. Novel prognostic biomarkers that are associated with tumor-infiltrating immune cells are required.

Our previous study indicated that lymphocyte cytosolic protein 2 (*LCP2*) protein tightly interacted with UBASH3B protein, which was identified as a novel prognostic biomarker and correlated with immune cell infiltration in the tumor microenvironment^6^. *LCP2* is an adapter protein-encoding gene that is involved in T cell antigen receptor (TCR)-activated protein tyrosine kinase pathway^7^ and is essential for normal T-cell development and activation by serine phosphorylation^8^. *LCP2* is involved in mediating the second signal from CD28/B7 families to thus regulate TCR signaling pathways^9^. In addition to T cells, there are several studies detailing the function of *LCP2* in other immune cells. *LCP2* plays an important role in NK-cell mediated recognition of missing-self targets^10^ and positively regulates antigen-induced mast cell activation by recruiting BCR^11^. Quantitative reductions of *LCP2* trigger immune dysregulation with the excessive production of proinflammatory cytokines and autoantibodies^12^. These studies indicate that *LCP2* deeply participates in immune responses through the regulation of immune cells. The function of *LCP2* might vary from diseases, and further studies are required according to the gene profile alteration of immune cells in the tumor microenvironment. With this regard, deciphering the role of *LCP2* in melanoma and the interaction between *LCP2* and immune cells in melanoma will be of great interest.

In our study, we used bioinformatic methods to present a schematic diagram of *LCP2* in skin cutaneous melanoma (SKCM). The highly expressed *LCP2* in metastatic skin cutaneous melanoma (SKCM-Metastasis) suggested it was associated with good clinical outcomes. Differential *LCP2* expression levels were able to affect the infiltration levels of immune cells. Furthermore, molecules of TCR signaling downstream and B7-CD28 family members were correlated with *LCP2* expression in metastatic skin cutaneous melanoma. Based on our findings, we identified *LCP2* as a prognostic biomarker for progressive-free survival (PFS) of patients with metastatic melanoma receiving anti-PD-1 immunotherapy. This study provides new insights into further exploration of the potential function and the mechanism of *LCP2* in anti-tumor immunity. Understanding the complex interplay of *LCP2* and the immune cells in melanoma could predict the therapeutic response of immune checkpoint-related therapy.

## Results

### *LCP2* has different expression level in Pan-cancer

**Figure 1:**
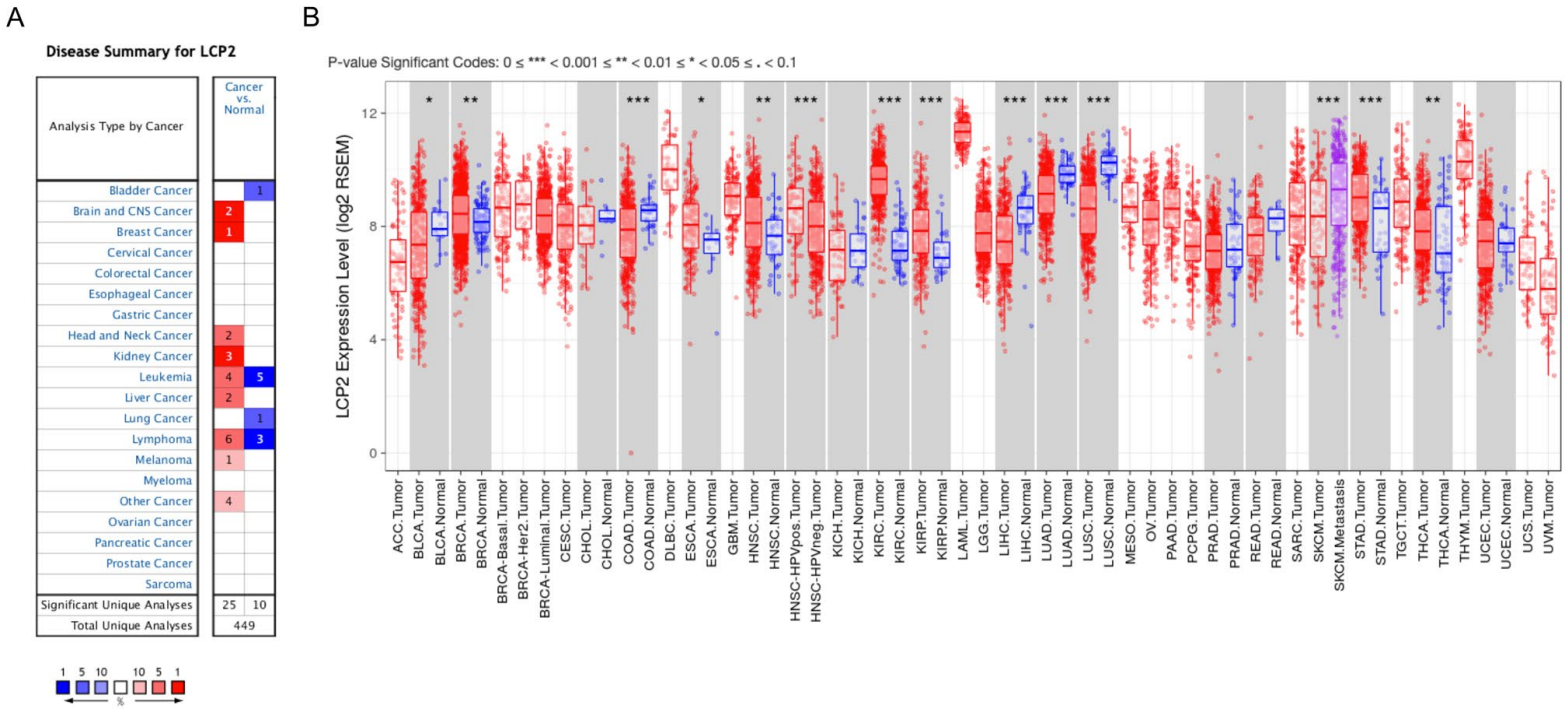
The gene expression of *LCP2* in different types of cancers. (**A**) Oncomine database showed *LCP2* was expressed in 11 types of cancers. (**B**) TIMER database displayed *LCP2* was expressed in multiple cancers based on TCGA database. *TCGA* The Cancer Genome Atlas, *TIMER* Tumor IMmune Estimation Resource, *BRCA* breast cancer, *ESCA* esophageal cancer, *KIRC* kidney renal clear cell cancer, *KIRP* kidney renal papillary cell cancer, *STAD* stomach adenocarcinoma, *THCA* thyroid cancer, *BLCA* bladder cancer, *COAD* colon adenocarcinoma, *LIHC* liver hepatocellular cancer, *LUAD* lung adenocarcinoma, *LUSC* lung squamous cell cancer, *HNSC* head and neck squamous cell cancer, *SKCM* skin cutaneous melanoma.

To explore the expression of *LCP2* in malignant tumors, we first investigated the Oncomine database, which contains abundant microarray data of GEO and TCGA database. In comparison with normal tissue, there were 25 studies showing that *LCP2* was upregulated in cancer, whereas 10 studies reporting downregulated *LCP2* in cancer (Fig. 1A). *LCP2* expression in the TIMER database including 29 types of tumors was sequentially evaluated. In comparison with corresponding normal tissues, *LCP2* was upregulated in 7 types of cancers, while downregulated in 5 types of cancers (Fig. 1B). Furthermore, HPV-positive head and neck cancer (HNSC-HPV positive) had higher *LCP2* expression than HPV-negative head and neck cancer. *LCP2* expression in metastatic skin cutaneous melanoma (SKCM-Metastasis) was higher than primary skin cutaneous melanoma (SKCM-Primary) (Fig. 1B).

### High expression of *LCP2* in metastatic skin cutaneous melanoma indicates a good prognosis

**Figure 2:**
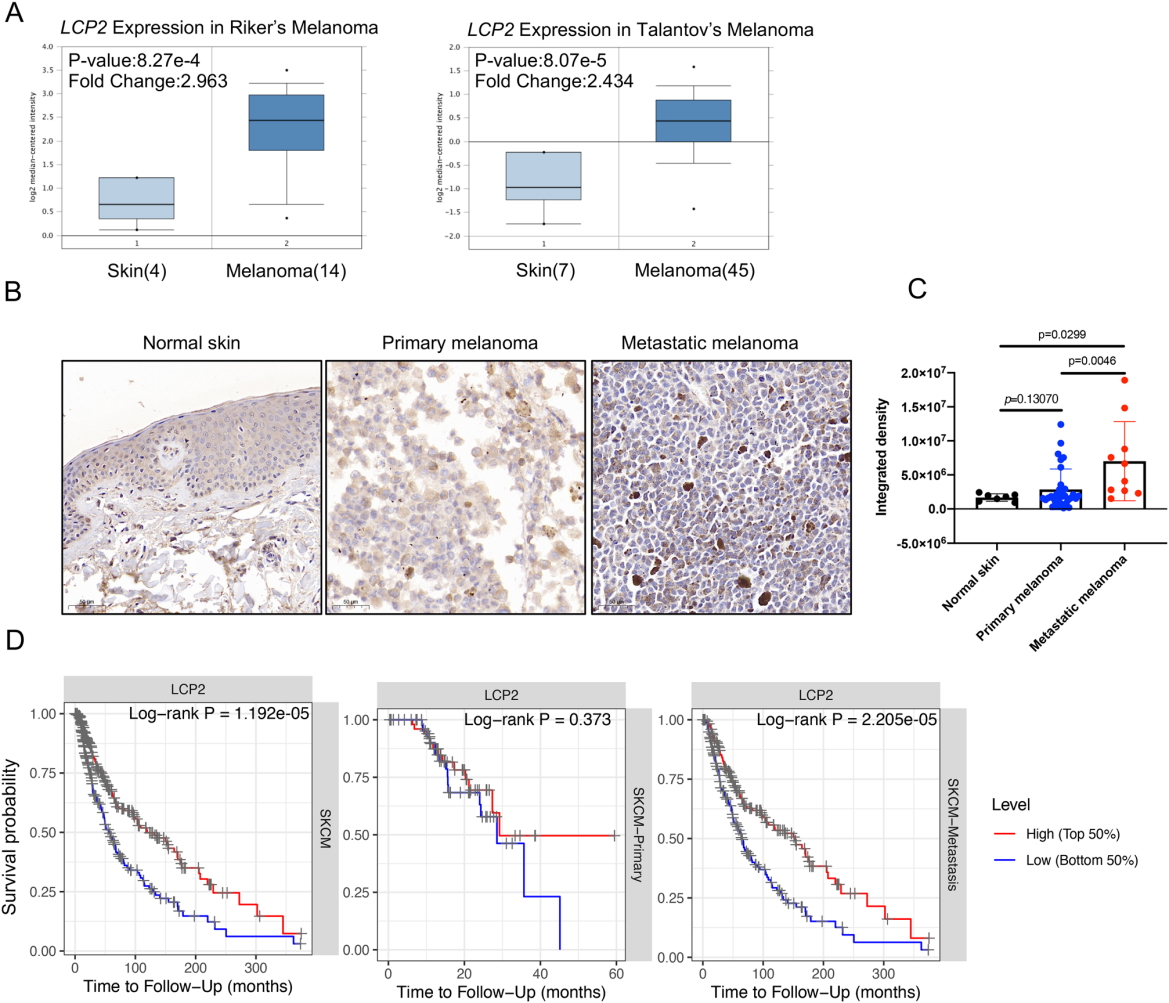
The expression and prognostic value of *LCP2* in melanoma. (**A**) *LCP2* expression level in human normal skin tissue and skin cutaneous melanoma. Based on the Oncomine database, box-plot diagrams were displayed to compare the *LCP2* level in human normal skin tissue with that in skin cutaneous melanoma from studies reported by Riker et al. (*p* value: 8.27e−4, Fold Change: 2.963) and Talantov al. (*p* value: 8.07e−5, Fold Change: 2.434). (**B**) LCP2 protein expression in normal skin, primary melanoma and metastatic melanoma tissue. (**C**) Quantitative analysis of immunohistochemistry indicated that LCP2 protein was highly expressed in metastatic melanoma tissue compared with normal skin and primary melanoma. (**D**) Based on differential *LCP2* expression level, patients with high-*LCP2* expression had a better prognosis as compared with those with low-*LCP2* expression in skin cutaneous melanoma and in cutaneous melanoma with metastasis, but not in primary sites of skin cutaneous melanoma.

**Figure 3:**
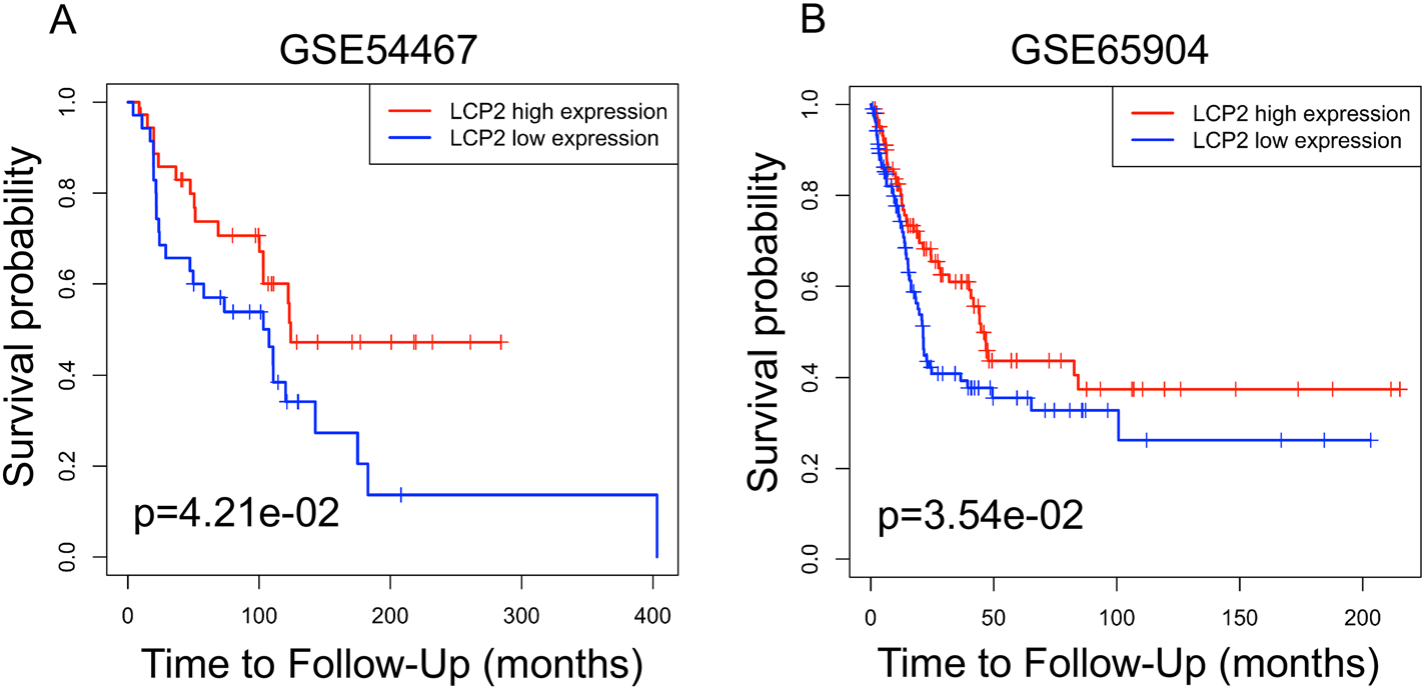
Prognostic value of LCP2 in melanoma. Kaplan–Meier curves display survival fraction for different *LCP2* expression groups of patients based on GSE54467 (**A**) and GSE65904 (**B**) datasets.

To explore the most obvious correlation between *LCP2* and overall survival, survival analysis was performed in TCGA Pan-cancer. We found that *LCP2* expression was significantly correlated with the overall survival in 9 cohorts (SKCM, SKCM-Metastasis, UVM, LGG, UCEC, THYM, SARC, GBM, HNSC-HPV positive). Among these cohorts, SKCM cohort exhibited the strongest correlation between *LCP2* expression and overall survival (Supplementary Table S1). Therefore, we focused on investigating the role of *LCP2* in the SKCM cohort, especially in the SKCM-Metastasis metastatic cohort. In comparison with normal skin, *LCP2* was upregulated in skin cutaneous melanoma in Riker’s study^13^ (*p* value: 8.27 × 10^−4^; Fold Change: 2.963) and in Talantov’s study^14^ (*p* value: 8.07 × 10^−5^; Fold Change: 2.434) (Fig. 2A). Quantitative analysis of immunohistochemistry indicated that LCP2 protein was also highly expressed in metastatic melanoma tissue compared to normal skin and primary skin cutaneous melanoma (Fig. 2B,C). To investigate the prognostic value of *LCP2* in the cohort, in which patients were received pharmacotherapy and radiation, TCGA-SKCM, GSE54467 and GSE65904 were included in this study. Kaplan–Meier curves showed that high *LCP2* expression was significantly associated with a good overall survival in the SKCM and the SKCM-Metastasis cohorts, but not significant in the SKCM-Primary cohort (Fig. 2D). Based on two metastatic melanoma datasets (GSE54467^15^ and GSE65904^16^), the prognostic value of *LCP2* expression was consistent with the result from the SKCM-Metastasis cohort (Fig. 3A,B).

Based on UALCAN analysis, we investigated the relationship between the clinical characteristics and *LCP2* expression. There were significant differences of *LCP2* expression in the different SKCM cohorts (primary vs. metastasis, *p* = 1.94 × 10^−8^) and individual cancer stages (stage 1 vs. stage 2, *p* = 2.42 × 10^−3^; stage 2 vs. stage 3, *p* = 1.97 × 10^−5^) (Supplementary Figure S1).

### *LCP2* and *LCP2*-correlated genes are involved in a variety of processes of immune response

**Figure 4:**
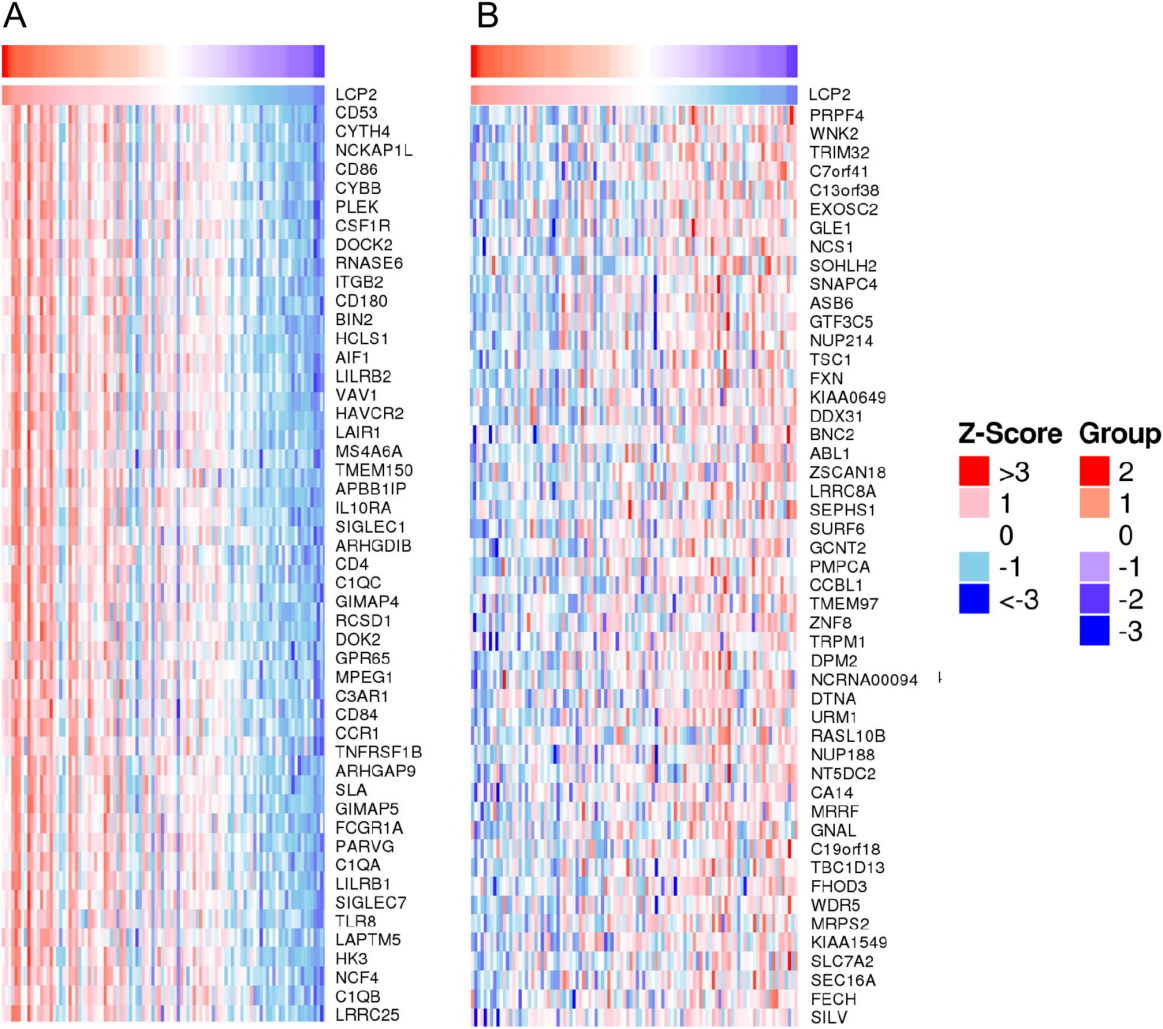
Heatmaps of *LCP2*-correlated genes. (**A**) Heatmap of top 50 positively correlated genes. (**B**) Heatmap of top 50 negatively correlated genes.

To further explore the genes interacting with *LCP2*, LinkedOmics analysis was performed. Top 10 genes that were positively correlated with *LCP2* were *CD53*, *CYTH4*, *NCKAP1L*, *CD86*, *CYBB*, *PLEK*, *CSF1R*, *DOCK2*, *RNASE6* and *ITGB2*. Top 10 genes that were negatively correlated with *LCP2* were *PRPF4*, *WNK2*, *TRIM32*, *C7orf41*, *C13orf38*, *EXOSC2*, *GLE1*, *NCS1*, *SOHLH2* and *SNAPC4*. Heatmaps of top 50 *LCP2*-positively correlated genes and *LCP2-*negatively correlated genes are displayed in Fig. 4A,B, respectively. These *LCP2*-correlated genes, such as *CD86*, *HAVCR2* and *SIGLEC1* were involved in multiple immune responses.

**Figure 5:**
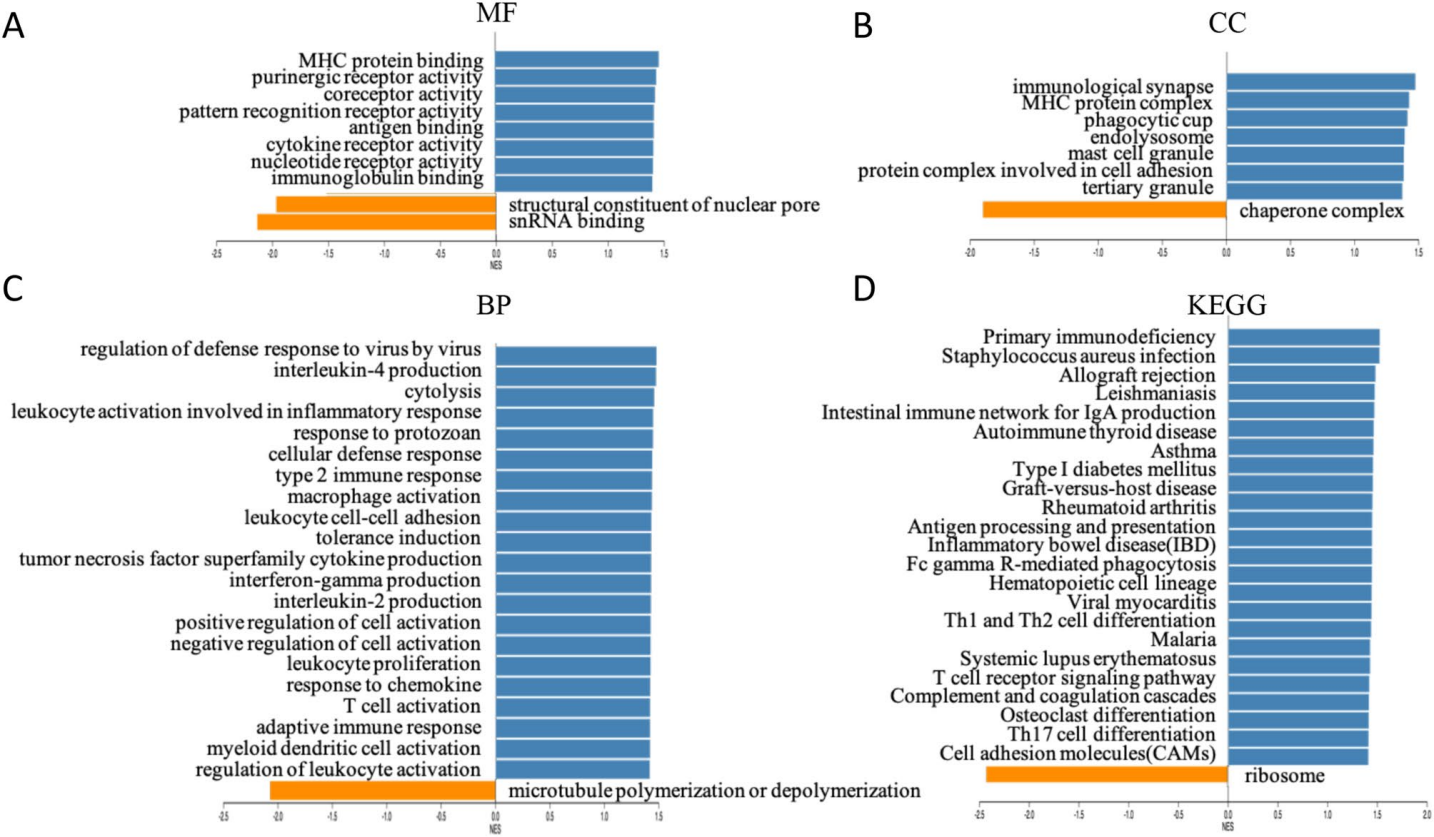
*LCP2*-correlated genes were involved in the GO and KEGG pathways. Based on the expression of *LCP2* in melanoma, GSEA analysis was performed and the enrichments of MF (**A**) CC (**B**), BP (**C**) and KEGG signaling pathways (**D**) were shown. *MF* molecular function, *CC* cellular component, *BP* biological process.

To further understand the functional enrichment of *LCP2*-correlated genes, GSEA method was used to analyze GO and KEGG signaling pathways. The major molecular function was located in “MHC protein binding” (Fig. 5A). In the cellular component, the highest normalized enrichment score was “immunological synapse” (Fig. 5B). In the biological process, “regulation of defense response to virus by virus” had the largest normalized enrichment score, with the maximum enrichment (Fig. 5C). For KEGG signaling pathways, several main pathways were enriched, including “antigen processing and presentation”, “Fc gamma R-mediated phagocytosis”, “Th1 and Th2 cell differentiation”, “T cell receptor signaling pathway” and “Th17 cell differentiation” (Fig. 5D). Collectively these results suggest *LCP2* plays a critical role in signal transmission of immune responses.

### *LCP2* is positively correlated with TCR signaling activation and B7/CD28 immune checkpoint families in metastatic melanoma

**Figure 6:**
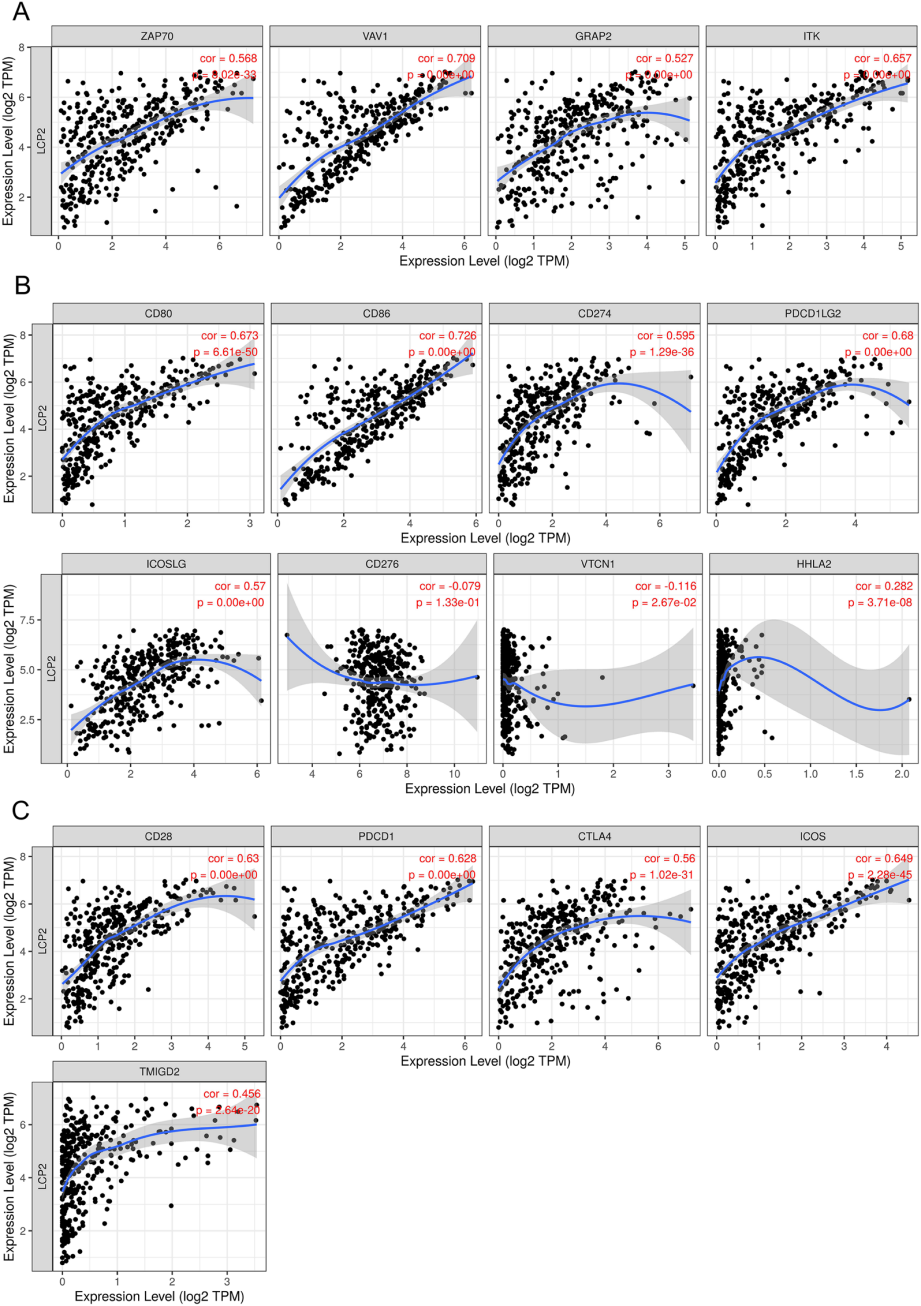
The correlation between *LCP2* and signal molecules of TCR/CD28-B7 checkpoint families. (**A**) *LCP2* expression level was correlated with *ZAP70*, *VAV1*, *GRAP2*, *ITK* in SKCM-Metastasis. (**B**) *LCP2* expression level was correlated with *CD80, CD86, CD274, PDCD1LG2, ICOSLG, CD276, VTCN1, HHLA2* in the SKCM-Metastasis cohort. (**C**) *LCP2* expression level was correlated with *CD28, CTLA4, PDCD1, ICOS* and *TMIGD2* in the SKCM-Metastasis cohort. *SKCM* skin cutaneous melanoma.

*LCP2* is an important gene which is directly involved in the activation of TCR signaling and indirectly affects TCR signaling through CD28 and B7 families^9^. We investigated the correlation between *LCP2* and recruited TCR signaling genes (*ZAP70*, *VAV1*, *GRAP2*, *ITK*). The results showed positive correlations between *LCP2* and *ZAP70* (cor = 0.568, *p* =  < 0.001), *LCP2* and *VAV1* (cor = 0.709, < 0.001), *LCP2* and *GRAP2* (cor = 0.527, < 0.001), *LCP2* and *ITK* (cor = 0.657, *p* < 0.001) (Fig. 6A). Based on T cell co-signaling pathway (ligand–receptor interactions in PathCards, https://pathcards.genecards.org), 35 out of 58 immune checkpoint genes were revealed in the *LCP2*-correlated gene sets (Supplementary Table S2). These results suggested that *LCP2* had a potentially correlated expression pattern with immune checkpoints. When studying B7 and CD28 immune checkpoint families, *LCP2* expression was positively correlated with *CD80* (cor = 0.673, *p* < 0.001), *CD86* (cor = 0.726, *p* < 0.001), *CD274* (cor = 0.595, *p* < 0.001), *PDCD1LG2* (cor = 0.68, *p* < 0.001), *ICOSLG* (cor = 0.57, *p* < 0.001), *HHLA2 *(cor = 0.282, *p* < 0.001), *CD28* (cor = 0.63, *p* < 0.001), *CTLA4* (cor = 0.56, *p* < 0.001), *PDCD1* (cor = 0.628, *p* < 0.001), *ICOS* (cor = 0.649, *p* < 0.001), and *TMIGD2* (cor = 0.456, *p* < 0.001) in SKCM-Metastasis cohorts. Whereas *LCP2* was negatively correlated with *VTCN1* (cor = -0.116, *p* = 0.0267) in SKCM-Metastasis cohorts but was not significantly correlated with *CD276* (cor = -0.079, *p* = 0.133) (Fig. 6B,C). Further analysis revealed that *LCP2* was positively correlated with other immune exhausting genes and immunosuppressive genes (*TIM3* also called *HAVCR2*, *LAG3*, *TOX*, *TIGIT*) (Supplementary Figure S2).

### *LCP2* is mainly correlated with tumor-infiltrating CD8^+^ T cells in metastatic melanoma

**Figure 7:**
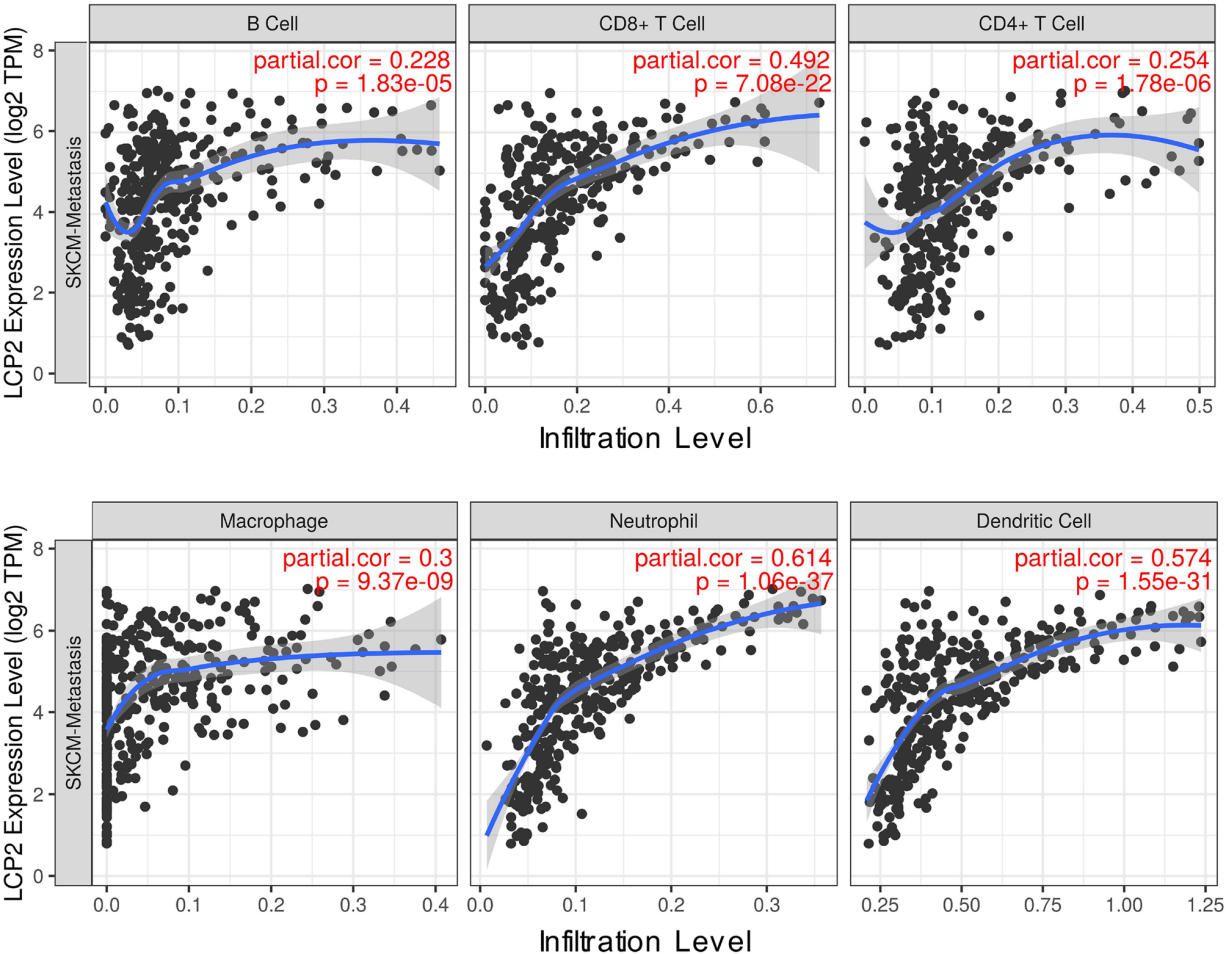
The correlation between *LCP2* and tumor-infiltrating immune cells. *LCP2* expression was correlated with tumor-infiltrating B cell, CD8^+^ T cell, CD4^+^ T cell, macrophage, neutrophil and dendritic cell in the SKCM-Metastasis cohort.

**Figure 8:**
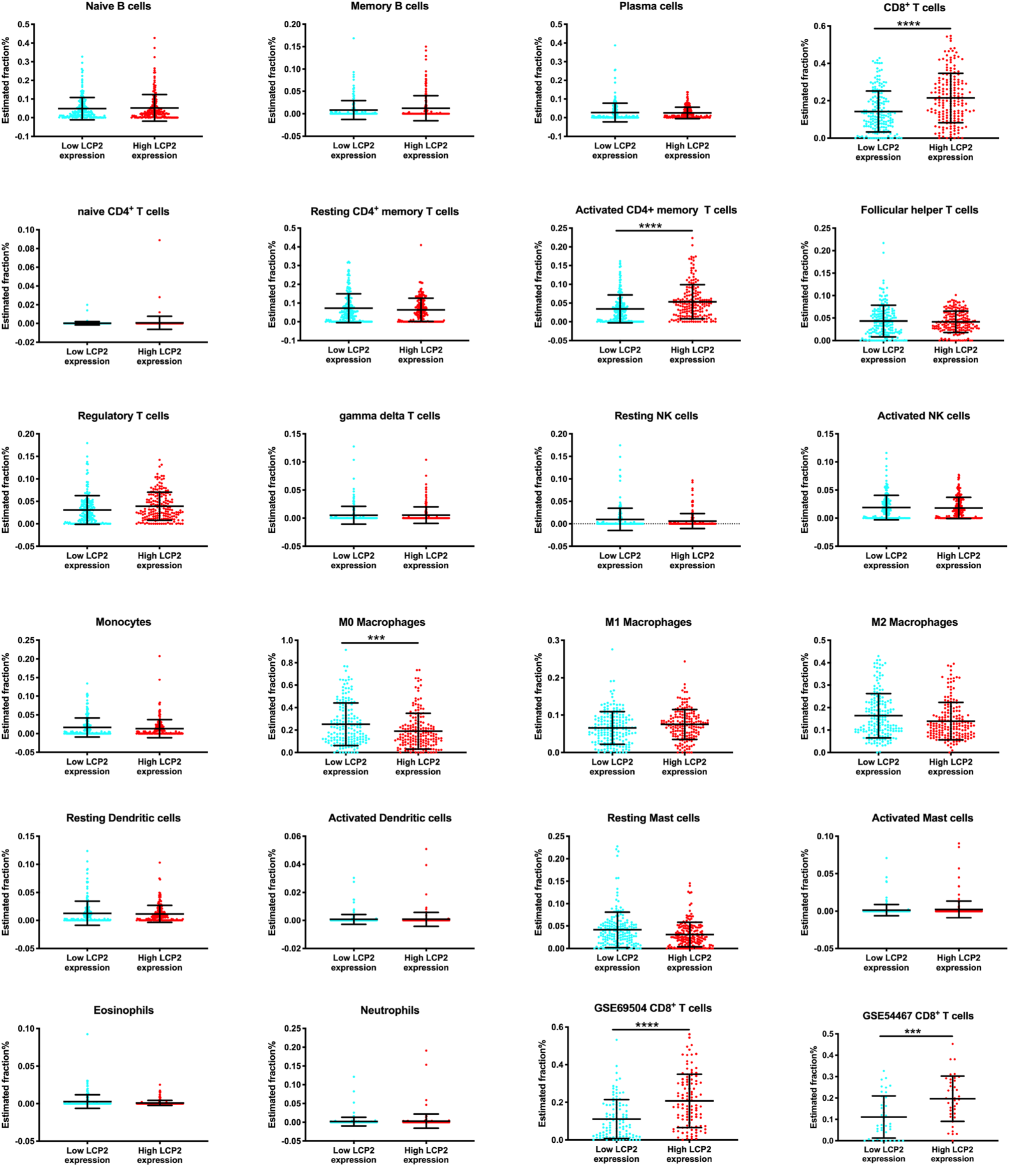
CIBERSORT analysis of 22 immune cells based on *LCP2* expression level. Significantly higher estimated fractions of CD8^+^ T cells and activated memory CD4^+^ T cells occurred in the high-*LCP2* expression group, whereas lower estimated fractions of M0 macrophages existed in the high-*LCP2* expression group. Validation cohorts (GSE65904 and GSE54467) indicated that significantly higher estimated fractions of CD8^+^ T cells occurred in the high-*LCP2* expression group.

The correlations between *LCP2* expression and 6 types of melanoma-infiltrating-immune cells were evaluated via TIMER. *LCP2* expression was correlated with tumor-infiltrating B cell, CD8^+^ T cell, CD4^+^ T cell, macrophage, neutrophil and dendritic cell in SKCM-Metastasis cohorts (Fig. 7). To further investigate more immune cell subtypes, gene signature file LM22 which defined 22 immune cell subtypes was applied and CIBERSORT was performed. Based on SKCM-Metastasis, there were significantly higher estimated fractions of CD8^+^ T cells and activated CD4^+^ T cells in the high-*LCP2* expression group, whereas lower estimated fractions of M0 macrophages in the high-*LCP2* expression group (Fig. 8). In the validation cohort of GSE65904 dataset, more CD8^+^ T cells were infiltrated in the high-*LCP2* expression group (Fig. 8), a similar infiltration pattern of CD8^+^ T cells was observed in the second validation cohort of GSE54467 dataset (Fig. 8). Although the infiltration of more CD8^+^ T cells in the high-*LCP2* expression group was not significantly observed in the TCGA-SKCM primary melanoma cohort, the trend observed was similar with metastatic melanoma (Supplementary Figure S3).

### *LCP2* acts as a novel prognostic biomarker of progression-free survival for patients who received anti-PD1 immunotherapy

**Figure 9:**
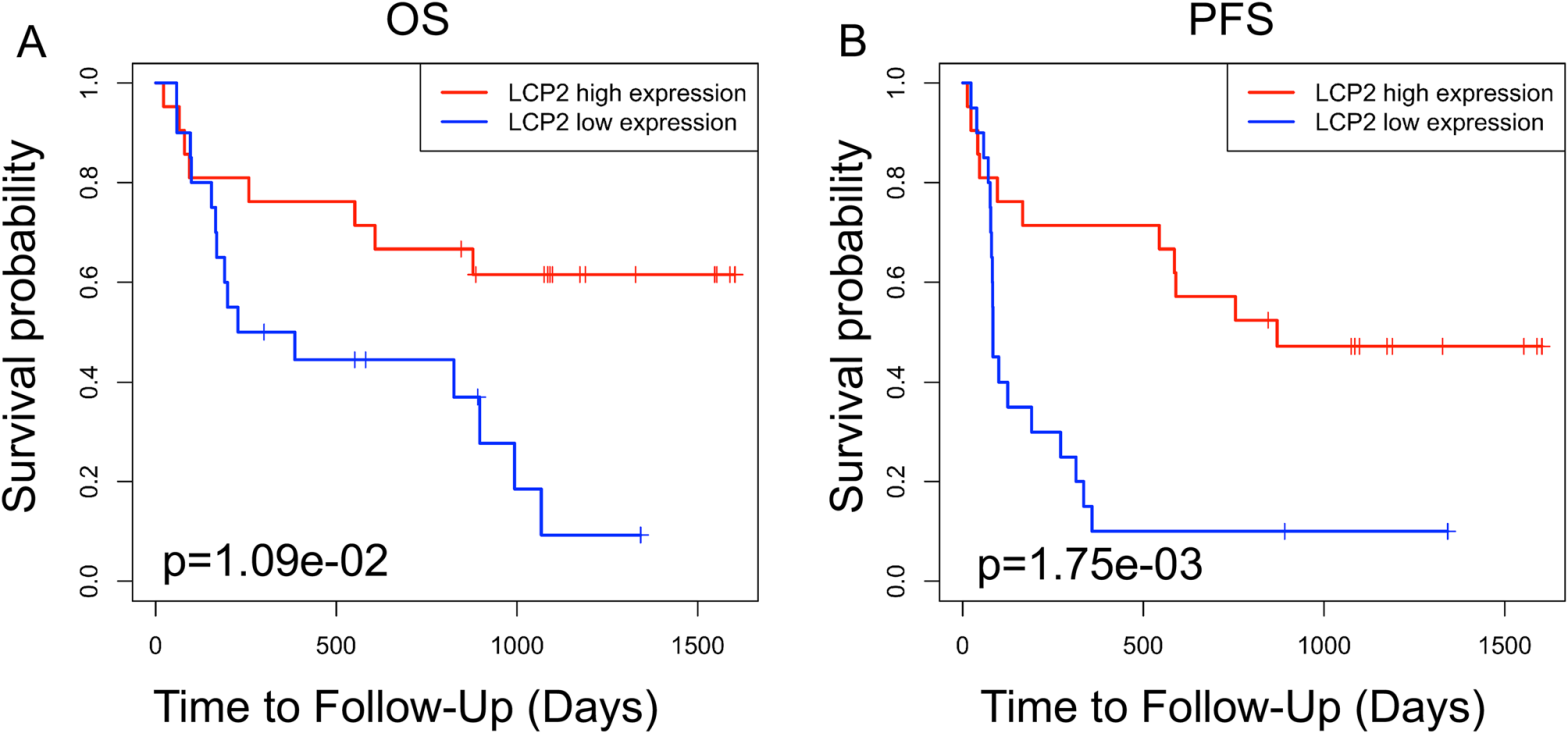
Prognostic value of LCP2 in melanoma patients receiving immune checkpoint blockade. Kaplan–Meier curves of OS (**A**) and PFS (**B**) for patients with low and high expression of *LCP2* in the Gide2019_PD1 cohort who received anti-PD-1 immunotherapy. *OS* overall survival, *PFS* progression-free survival.

To further explore the prognostic value of *LCP2* in patients who received immune checkpoint inhibitors, the Gide2019_PD1 and Gide2019_PD1+CTLA4 cohorts were included in this study^17^. Kaplan–Meier curves of overall survival and progression-free survival were plotted in these two cohorts. In the Gide2019_PD1 cohort of patients receiving anti-PD-1 immunotherapy, we found that high-*LCP2* expression was associated with a good overall survival (Fig. 9A, *p* = 1.09 × 10^−2^) and progression-free survival (PFS) (Fig. 9B, *p* = 1.75 × 10^−3^). To strengthen results obtained in Kaplan–Meier analysis, survival (PFS and OS) data were also evaluated by univariate and multivariate Cox regression analyses, preferably using *LCP2* expression as continuous variable to avoid possible bias due to cutoff point selection. Tumor-infiltrating immune cells were analyzed with *LCP2* expression as variables. The results indicated that LCP2 was a prognostic factor of progression-free survival (*p* = 0.01) (Supplementary Table 3), but not overall survival (*p* = 0.072) (Supplementary Table 4). In addition, *LCP2* expression was unable to predict the overall survival (*p* = 0.511) and progression-free survival (*p* = 0.150) in the Gide2019_PD1 + CTLA4 cohort of patients receiving anti-PD-1 and anti-CTLA4 combination immunotherapy^17^ (Supplementary Figure S4).

## Discussion

In the present study, we analyzed RNA-seq data by using multiple tools and performed immunohistochemistry of tissue microarray to investigate the expression of LCP2 in melanoma. We found mRNA and protein of *LCP2* was highly expressed in SKCM-Metastasis. The high expression of *LCP2* was associated with good survival of patients in SKCM-Metastasis cohort, in which patients received pharmacotherapy and radiation. Furthermore, *LCP2* was involved in some immune-responses-related signaling pathways and high expression of *LCP2* increased the infiltration of anti-tumor immune cells and thus helped to predict the progression-free survival of patients with metastatic skin cutaneous melanoma receiving anti-PD-1 immunotherapy.

LCP2 protein plays an important role in the TCR-mediated signal transduction pathway and tumor malignancy. High LCP2 protein expression is correlated with aggressive behavior in chronic lymphocytic leukemia cells^18^. *LCP2* is involved in colon cancer metastasis as a differentially expressed gene^19^ and plays a critical role in inflammation of colorectal cancer^20^. The high *LCP2* expression pattern also occurred in metastatic skin cutaneous melanoma and was correlated with good overall survival. In addition, LCP2 protein tightly interacted with UBASH3B protein, which was identified as a novel prognostic biomarker and correlated with immune cell infiltration in the tumor microenvironment^6^. The function of LCP2 protein in normal T cell differentiation and activation had been reported that serine phosphorylation of LCP2 protein was essential to T cell differentiation by modulating Th1 and Th2 cells function^8^. GSEA indicated *LCP2* was correlated with “T cell receptor signaling pathway”, “Th1 and Th2 cell differentiation” and “Th17 cell differentiation”. These results were consistent with previous studies^8^. Although mislocalization of LCP2 protein caused CD4^+^ T cells to skew towards the inflammatory Th1 and Th17 lineages^21^, the role of *LCP2* in Th17 cell differentiation is still unclear and is worth investigating.

Activation of the TCR signaling pathway is important for anti-tumor immune response. In this study, *LCP2* was found to be associated with TCR signal molecules. These results are in accordance with recent studies indicating that *LCP2* was involved in TCR-mediated signal transduction^22^. Additionally, the receptor-ligand interactions of immune checkpoints regulated TCR signal transduction and affected anti-tumor immune response^23^. Interestingly, we found a few immune checkpoint genes that were correlated with *LCP2*. The suppressive immune checkpoints and “exhausting genes” regulated functions of immune cells in the tumor microenvironment, resulting in CD8^+^ T cells dysfunction^24^. In addition, a large number of studies have reported the different roles of various tumor-infiltrating immune cells in melanoma^2,24–26^. In the SKCM metastasis cohort, the increase in the amount of CD8^+^ T cells and activated CD4^+^ memory T cells may have more potential to kill cancer cells after undergoing anti-PD-1 immunotherapy. High-*LCP2* expression patients in our study had more infiltrations of CD8^+^ T cells, which was associated with a good prognosis^27^. Kaplan–Meier Curve indicated that *LCP2* was associated with a good overall survival of patients with skin cutaneous metastatic melanoma, who did not receive immunotherapy. It is suggested that *LCP2* may serve as a favorable prognostic biomarker and played an important role in skin cutaneous metastatic melanoma through the main function of CD8^+^ T cells. Excitingly, *LCP2* was a good prognostic biomarker of progression-free survival for patients who received anti-PD1 immunotherapy.

There were some limitations in this study. First, the potential mechanisms of *LCP2* involving anti-PD1 immunotherapy and combination immunotherapy are still unclear. Second, the interactions between *LCP2* and immune checkpoints are required by further analysis in melanoma.

In summary, our results offer new insights into the role of *LCP2* associated immune responses in metastatic skin cutaneous melanoma. The integrated analysis of *LCP2* expression datasets and the utilization of multiple methods help us translate bioinformatics data into biomarkers identification for clinical application in the prognostic prediction of melanoma. Altogether our results established a novel role of *LCP2* as an immune regulator in melanoma and identified *LCP2* as a rational prognostic biomarker in patients with metastatic skin cutaneous melanoma receiving anti-PD-1 immunotherapy.

## Methods

### Expression datasets and clinical information

RNA transcriptome data of all melanoma samples (n = 472) (SKCM), primary skin cutaneous melanoma (SKCM-Primary) (n = 104) and metastatic SKCM (SKCM-Metastasis) (n = 368) were downloaded from TCGA (https://portal.gdc.cancer.gov/).

Transcriptome and clinical survival data of validation cohorts (GSE54467 (n = 71) and GSE65904 (n = 210)) were included in this study. Raw data were normalized by log(X + 1) for further analysis.

2 cohorts of metastatic melanoma with immunotherapy (Gide2019_PD1 (n = 41), Gide2019_PD1 + CTLA4 (n = 32)) were included to evaluate the prediction ability of *LCP2*.

### Expression analysis

The mRNA expression levels of *LCP2* in different types of cancer were analyzed in the Oncomine database (https://www.oncomine.org) and mRNA expression level of *LCP2* in skin cutaneous melanoma and normal skin tissue was investigated in a serial of melanoma studies, including Riker and Talantov’s studies.

This study was approved by the Clinical Research Ethics Committee of the Second Xiangya Hospital of Central South University. Immunohistochemistry (IHC) was used to evaluate the expression of LCP2 protein. The protocol of immunohistochemistry was detailed in the previous study^6^. The anti-LCP2 antibody (#DF7020, Biosciences, China) was used as the primary antibody. A human melanoma tissue microarray (K063Me01, Bioaitech, China) which contained 63 samples was purchased from commercial company and used in the study. Our inclusive criteria were normal skin and skin cutaneous melanoma. 7 normal skin, 32 primary melanoma and 10 metastatic melanoma samples were included as three groups and an IHC Toolbox plug-in was used to quantitative the brown positive particles. The integrated density of the brown positive particles was used to compare the expression of LCP2 protein in different groups.

### UALCAN analysis

UALCAN database (http://ualcan.path.uab.edu/index.html)^28^ is allowed to analyze the relationship between relative expression of *LCP2* and different types of skin tissue (normal skin tissue, primary skin cutaneous melanoma and metastatic skin cutaneous melanoma), as well as clinical characteristics of melanoma patients, such as age, gender, stages, weight and promoter methylation level.

### LinkedOmics analysis

LinkedOmics (http://www.linkedomics.org)^29^ is a publicly available portal that includes multi-omics data from all 32 TCGA Cancer types. We used RNA-seq data of SKCM and chose the Pearson test to calculate the correlation coefficient for *LCP2* correlated significant genes. In the LinkInterpreter module, Gene set enrichment analysis (GSEA) was applied to generate Gene Ontology (GO) and Kyoto Encyclopedia of Genes and Genomes (KEGG) pathways^30^. “Rank Criteria (from LinkFinder Result)” was FDR. “Minimum Number of Genes (Size)” was 3 and “Simulations” was 10,000.

### TIMER

The different mRNA expression of LCP2 was analyzed in all types of cancers in Tumor IMmune Estimation Resource (TIMER, https://cistrome.shinyapps.io/timer)^31^ and three melanoma cohorts: SKCM, SKCM-Primary, SKCM-Metastasis were used to evaluate the association between *LCP2* mRNA expression level and overall survival. In the SKCM cohorts, patients with primary and metastatic skin cutaneous melanoma were included. The correlations between *LCP2* and immune cells (B cell, CD8^+^ T cell, CD4^+^ T cell, macrophage, neutrophil and dendritic cell) were evaluated. Furthermore, the correlations between *LCP2* and downstream signaling molecules of TCR and B7-CD28 immune checkpoint family members were studied.

### CIBERSORT

CIBERSORT^32^ is an online website for analyzing 22 immune cell types infiltrated in different kinds of cancers. Fragments per kilobase million (FPKM) data of SKCM-Metastasis were downloaded from the TCGA database and the data were extracted and merged by Perl software as a matrix file. FPKM value was converted to TPM value by R Studio Version 1.1.463. In addition, matrixes of GSE54467 and GSE65904 were downloaded and annotated by R studio. The matrix files were uploaded into the CIBERSORT network (https://cibersort.stanford.edu) as a Mixture file. LM22 was selected as “Signature gene file” and “Permutations” run 1000. Disable quantile normalization was only recommended for RNA-seq of SKCM-Metastasis. Deconvolution results were expressed as relative fractions and the analysis was performed in the SKCM-Primary, SKCM-Metastasis, GSE54467 and GSE65904 datasets, respectively. Based on the median expression of *LCP2* as a dynamical cutoff, melanoma patients were divided into two groups, high-*LCP2*-expression group and low-*LCP2*-expression group. Data were imported into GraphPad Prism 7 and the relative value of 22 types of immune cells was evaluated in both high- and low-*LCP2* expression groups.

### Kaplan–Meier curve analysis

The “survival” R package was used to plot the Kaplan–Meier curve. The median value of *LCP2* expression was settled as a cut-off. The patients were divided into two groups: high-*LCP2*-expression group and low-*LCP2*-expression group. Overall survival and progression-free survival were used to evaluate the prognostic value of *LCP2* expression in melanoma. GSE54467, GSE65904, Gide2019_PD1, Gide2019_PD1 + CTLA4 cohorts were used to evaluate the prognostic value of *LCP2*.

### Statistics analysis

The normally distributed quantitative data are shown as mean ± standard deviation (SD). The Student’s t-test is used to compare immune cell fractions in high- and low- *LCP2*-expression groups for statistical analysis. KM plot was used to evaluate the correlation between LCP2 and OS/PFS through the two-sided Wald test in a Cox proportional hazards regression analysis. A Bonferroni test was displayed as a correction for multiple comparisons. When *p* < 0.05, statistical differences are considered.

## Supplementary information

Supplementary information.

## Abbreviations

*LCP2*: Lymphocyte cytosolic protein 2
TCR: T cell antigen receptor
KIRC: Kidney renal clear cell cancer
KIRP: Kidney renal papillary cell cancer
SKCM-Metastasis: Skin cutaneous melanoma with metastasis
STAD: Stomach adenocarcinoma
THCA: Thyroid cancer
BLCA: Bladder cancer
COAD: Colon adenocarcinoma
LIHC: Liver hepatocellular cancer
HNSC: Head and neck squamous cell cancer
LUAD: Lung adenocarcinoma
LUSC: Lung squamous cell cancer
