# Supplementary material for: A novel prognostic biomarker LCP2 correlates with metastatic melanoma-infiltrating CD8+ T cells
Source: Sci Rep. 2021 Apr 28;11:9164. doi: 10.1038/s41598-021-88676-9 (PMC8080722; doi:10.1038/s41598-021-88676-9)
Supplement: Supplementary file 1 — Supplementary information. [file 41598_2021_88676_MOESM1_ESM.pdf]

A novel prognostic biomarker *LCP2* correlates with metastatic melanoma-infiltrating CD8$^+$ T cells

Zijun Wang[1, 2], Mou Peng[3, 4*]

[1] Institute of Dermatology, Chinese Academy of Medical Sciences and Peking Union Medical College, Nanjing, Jiangsu, China.

[2] Department of Dermatology, The Second Xiangya Hospital, Central South University, Changsha, Hunan, China.

[3] Department of Microbiology and Immunology, Albert Einstein College of Medicine, Bronx, New York.

[4] Department of Urology, The Second Xiangya Hospital, Central South University, Changsha, Hunan, China.

*Corresponding Author

Mou Peng

Department of Urology,

The Second Xiangya Hospital, Central South University

No.139 Renmin Middle Rd, Changsha, Hunan 410011, China

Tel: 86-731-85295134

Fax: 86-731-85295134

Email: pengmou@csu.edu.cn

Supplementary Figure S1 The relationship between LCP2 expression with clinical characteristics of cutaneous melanoma. A. LCP2 was highly expressed in SKCM with metastasis compared to primary SKCM (p=1.93E-08). B. There were no significant differences in patient's age in SKCM. C. LCP2 was over-expressed in SKCM with stage 1 and stage 3 compared to stage 2, respectively (stage 1 vs stage 2: P=2.422E-03, stage 2 vs stage 3: P=1.971E-05). D&E&F. There were no significant differences in patient's gender (D), weight (E) and promoter methylation level of LCP2 in SKCM (F). SKCM: skin cutaneous melanoma.

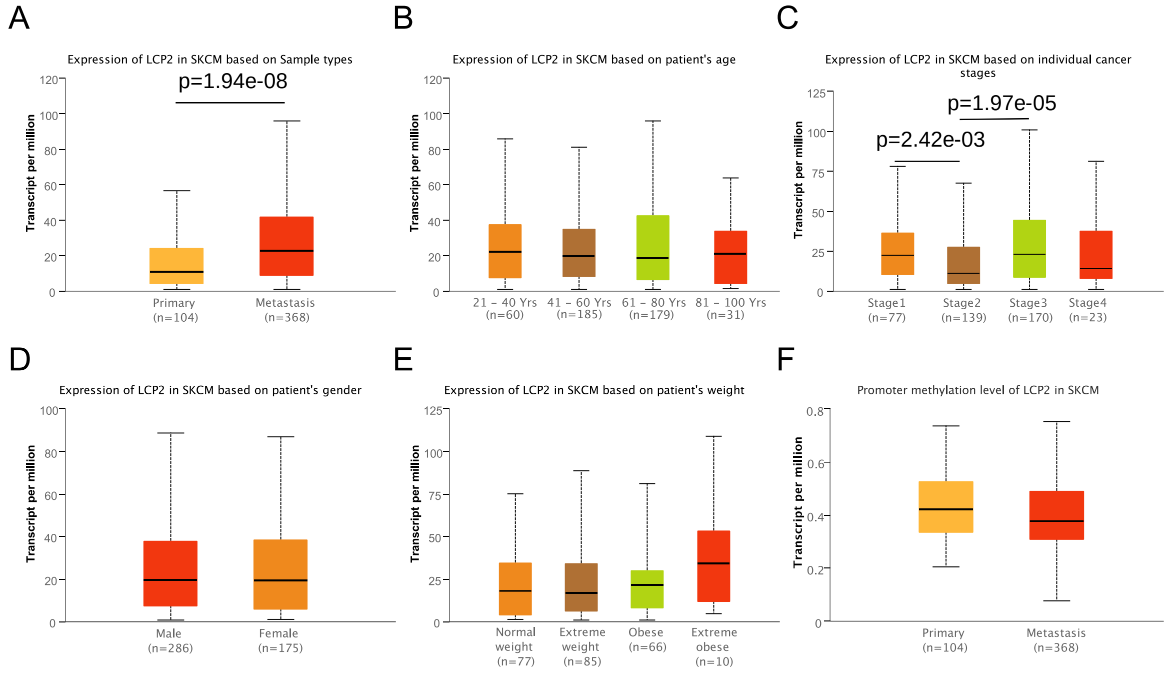

Supplementary Figure S2 The *LCP2* expression was positively correlated with *HAVCR2*, *LAG3*, *TOX* and *TIGIT* in metastatic cutaneous melanoma.

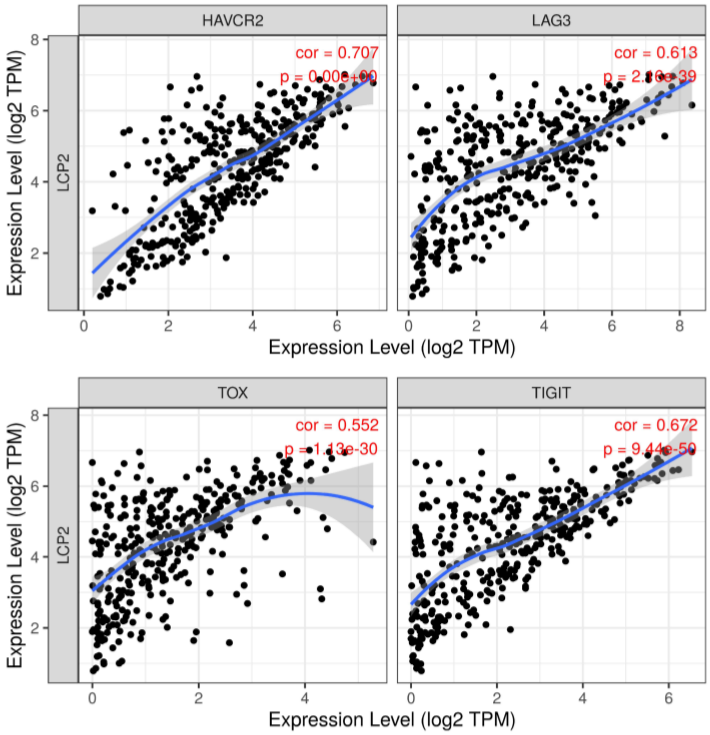

Supplementary Figure S3 CIBERSORT analysis of 22 immune cells based on *LCP2* expression level in primary melanoma cohort. Significantly lower estimated fractions of resting NK cells existed in the high-*LCP2* expression group.

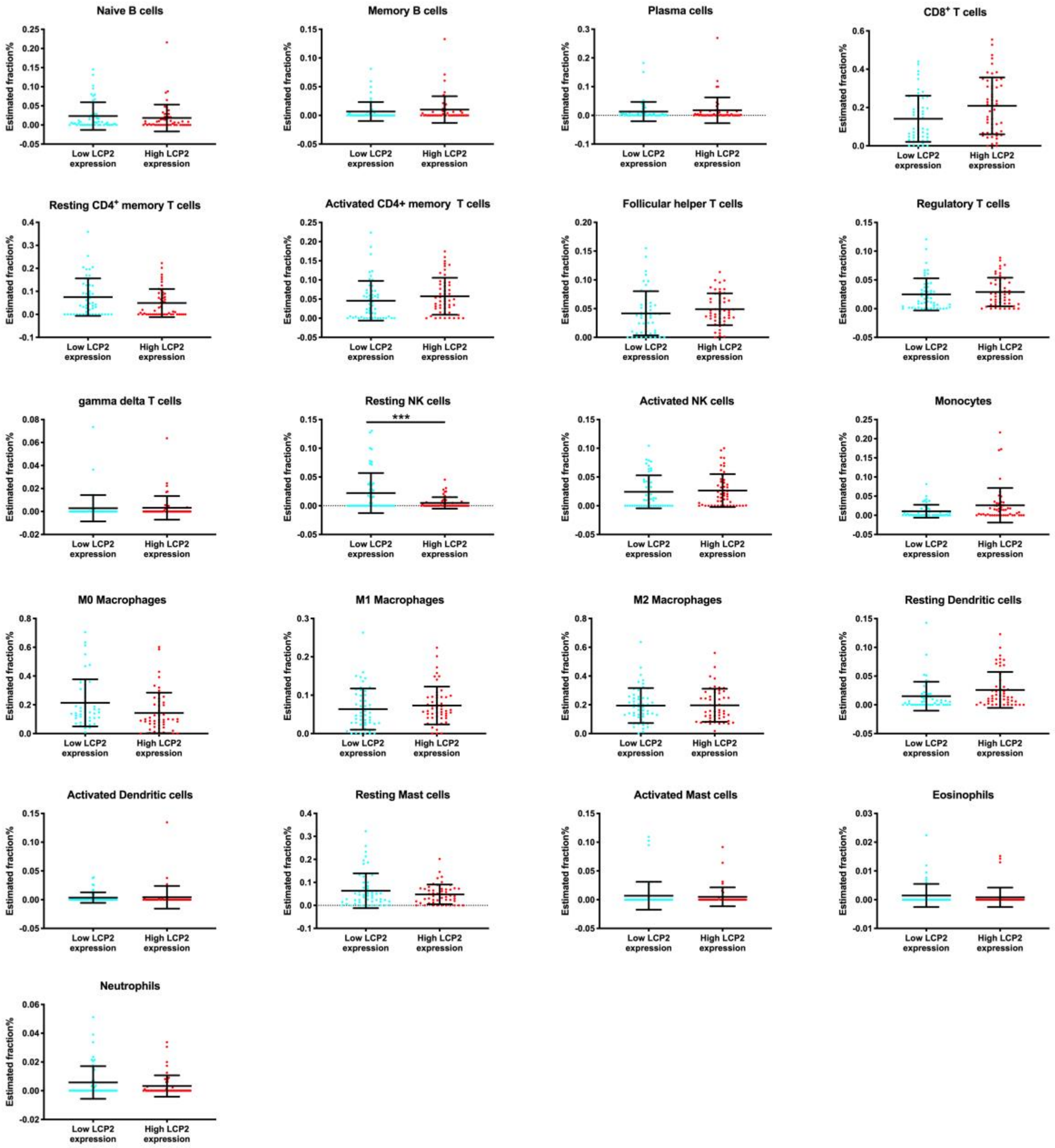

Supplementary Figure S4 Kaplan-Meier curves of OS and PFS for low- and high-*LCP2* expression patients after anti-PD1 immunotherapy from Gide2019_PD1+CTLA4 cohorts. OS: overall survival; PFS: progression-free survival.

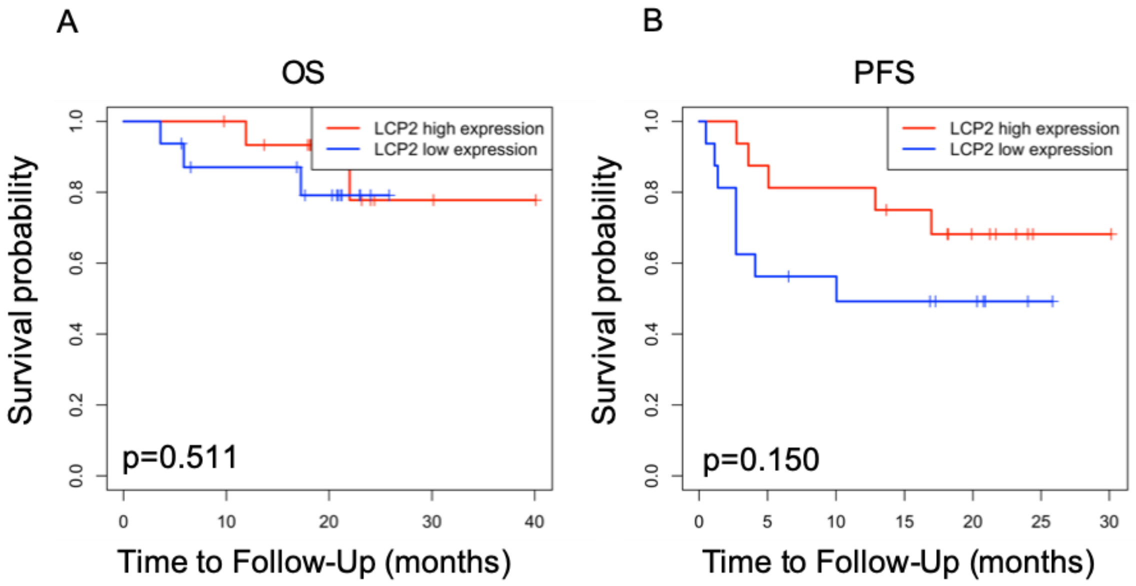

Supplementary Table S1 The correlation between LCP2 and overall survival in pan-cancers

cancer	variable	p-value
SKCM	LCP2	**1.192E-05**
SKCM-Metastasis	LCP2	**2.205E-05**
UVM	LCP2	**1.466E-04**
LGG	LCP2	**5.174E-03**
UCEC	LCP2	**9.151E-03**
THYM	LCP2	**9.737E-03**
SARC	LCP2	**0.013**
GBM	LCP2	**0.016**
HNSC-HPVpos	LCP2	**0.019**
THCA	LCP2	0.069
ESCA	LCP2	0.081
TGCT	LCP2	0.085
STAD	LCP2	0.118
READ	LCP2	0.160
BRCA-Her2	LCP2	0.168
LUSC	LCP2	0.182
CESC	LCP2	0.193
LUAD	LCP2	0.198
BRCA	LCP2	0.202
PCPG	LCP2	0.311
OV	LCP2	0.357
SKCM-Primary	LCP2	0.373
CHOL	LCP2	0.386
BRCA-Luminal	LCP2	0.400
PAAD	LCP2	0.413
KICH	LCP2	0.428
MESO	LCP2	0.505
HNSC	LCP2	0.552
DLBC	LCP2	0.637
KIRP	LCP2	0.665
BLCA	LCP2	0.719
COAD	LCP2	0.732
HNSC-HPVneg	LCP2	0.748
PRAD	LCP2	0.752
ACC	LCP2	0.812
UCS	LCP2	0.839
LIHC	LCP2	0.924
KIRC	LCP2	0.946
BRCA-Basal	LCP2	0.989

Supplementary Table S2 *LCP2* correlated T cell co-signaling pathway genes in metastatic melanoma.

LCP2 **correlated T cell co-signaling pathway genes**	**Correlation coefficient**	**p value**
HLA-DRA	0.711	5.50E-58
CD3E	0.656	0
CD80	0.673	6.61E-50
CD86	0.726	0
CD274	0.595	1.29E-36
HHLA2	0.282	3.71E-08
CD28	0.630	0
ICOS	0.649	2.28E-45
TMIGD2	0.456	2.64E-20
PDCD1LG2	0.680	0
PDCD1	0.628	0
BTLA	0.615	1.06E-39
BTNL8	0.532	2.70E-28
BTN3A1	0.513	0
CD48	0.687	0
SLAMF1	0.631	2.86E-42
CD2	0.689	0
CD244	0.572	2.64E-33
TIMD4	0.580	2.10E-34
HAVCR1	0.433	3.00E-18
LGALS9	0.603	0
HAVCR2	0.707	0
CD226	0.612	3.73E-39
TIGIT	0.672	9.44E-50
LAIR1	0.700	0
CD40	0.614	0
TNFSF14	0.643	2.77E-44
CD40LG	0.566	1.64E-32
CD70	0.555	4.08E-31
TNFSF8	0.667	1.03E-48
TNFSF18	0.333	5.61E-11
TNFRSF9	0.698	5.24E-55
CD27	0.638	1.76E-43
TNFRSF8	0.619	2.39E-40
TNFRSF18	0.586	0

Supplementary S3 Univariate and multivariate analysis for evaluating the prognostic factors of progression-free survival in the Gide2019_PD1 cohort

	univariate Cox analysis			multivariate Cox analysis		
Characteristics	Hazard Ratio	CI95	P. Value	Hazard Ratio	CI95	P. Value
B_cells_memory	0.01	0-2.08	0.088			
B_cells_naive	0.43	0.01-21.35	0.673			
Dendritic_cells_activated	0	0-116608.22	0.389			
Dendritic_cells_resting	616.56	0.09-4400411.87	0.156			
Eosinophils	0.84	0-242.86	0.953			
Macrophages_M0	0.23	0-35.89	0.571			
Macrophages_M1	0.02	0-28.22	0.297			
Macrophages_M2	5.38	0.19-150.69	0.323			
Mast_cells_activated	2.07	0.01-659.52	0.804			
Mast_cells_resting	48.83	2-1190.13	**0.017**	5.25	0.16-177.53	0.356
Monocytes	0.15	0-1905.58	0.696			
Neutrophils	0.28	0.01-13.45	0.52			
NK_cells_activated	2640.27	0.25-28153691.89	0.096			
NK_cells_resting	0	0-7.98	0.148			
Plasma_cells	0.23	0-20.91	0.52			
T_cells_CD4_memory_activated	0	0-0.77	**0.04**	0	0-2.07	0.081
T_cells_CD4_memory_resting	14.99	0.22-1001.97	0.207			
T_cells_CD4_naive	34.6	0.93-1281.24	0.054			
T_cells_CD8	0.01	0-0.73	**0.035**	1.07	0.01-92.45	0.977
T_cells_follicular_helper	0	0-0.07	**0.016**	0	0-1.12	0.052
T_cells_gamma_delta	0	0-6.71	0.098			
Tregs	0.2	0-227.46	0.657			
LCP2	0.7	0.53-0.92	**0.01**	0.98	0.68-1.41	0.923

Supplementary Table S4 Univariate and multivariate analysis for evaluating the prognostic factors of overall survival in the Gide2019_PD1 cohort

		univariate Cox analysis			multivariate Cox analysis	
Characteristics	Hazard Ratio	CI95	P. Value	Hazard Ratio	CI95	P. Value
B_cells_memory	0.09	0-21.91	0.391			
B_cells_naive	0.55	0.01-38.91	0.783			
Dendritic_cells_activated	0	0-220752.49	0.326			
Dendritic_cells_resting	0.2	0-11006.91	0.775			
Eosinophils	6.8	0.02-2303.2	0.519			
Macrophages_M0	0.37	0-108.18	0.729			
Macrophages_M1	0	0-2.36	0.066			
Macrophages_M2	5.84	0.15-229.55	0.346			
Mast_cells_activated	0.78	0-249.34	0.933			
Mast_cells_resting	52.33	1.72-1589.3	**0.023**	14.94	0.4-551.87	0.142
Monocytes	0.01	0-5233.32	0.492			
Neutrophils	0.41	0.01-24.41	0.67			
NK_cells_activated	3257.01	0.11-100517288.52	0.125			
NK_cells_resting	0	0-6.7	0.118			
Plasma_cells	0.37	0-50.11	0.691			
T_cells_CD4_memory_activated	0	0-1.69	0.072			
T_cells_CD4_memory_resting	28.34	0.22-3587.84	0.176			
T_cells_CD4_naive	10.35	0.2-527.33	0.244			
T_cells_CD8	0.02	0-1.45	0.072			
T_cells_follicular_helper	0	0-0.41	**0.037**	0	0-2.17	0.064
T_cells_gamma_delta	0	0-2.84	0.068			
Tregs	0.09	0-506.65	0.58			
LCP2	0.76	0.56-1.03	0.072			
